# Perturbations in growth trajectory due to early diet affect age‐related deterioration in performance

**DOI:** 10.1111/1365-2435.12538

**Published:** 2015-08-29

**Authors:** Who‐Seung Lee, Pat Monaghan, Neil B. Metcalfe

**Affiliations:** ^1^ Institute of BiodiversityAnimal Health and Comparative MedicineCollege of Medical, Veterinary and Life SciencesUniversity of GlasgowGraham Kerr BuildingGlasgowG12 8QQUK; ^2^ Present address: Southwest Fisheries Science CenteNOAA Fisheries and Center for Stock Assessment ResearchUniversity of CaliforniaSanta CruzCA95064USA

**Keywords:** ageing, compensatory growth, life history, nutrition, senescence, trade‐off

## Abstract

1. Fluctuations in early developmental conditions can cause changes in growth trajectories that subsequently affect the adult phenotype. Here, we investigated whether compensatory growth has long‐term consequences for patterns of senescence.
2. Using three‐spined sticklebacks (*Gasterosteus aculeatus*), we show that a brief period of dietary manipulation in early life affected skeletal growth rate not only during the manipulation itself, but also during a subsequent compensatory phase when fish caught up in size with controls.
3. However, this growth acceleration influenced swimming endurance and its decline over the course of the breeding season, with a faster decline in fish that had undergone faster growth compensation.
4. Similarly, accelerated growth led to a more pronounced reduction in the breeding period (as indicated by the duration of sexual ornamentation) over the following two breeding seasons, suggesting faster reproductive senescence. Parallel experiments showed a heightened effect of accelerated growth on these age‐related declines in performance if the fish were under greater time stress to complete their compensation prior to the breeding season.
5. Compensatory growth led to a reduction in median life span of 12% compared to steadily growing controls. While life span was independent of the eventual adult size attained, it was negatively correlated with the age‐related decline in swimming endurance and sexual ornamentation.
6. These results, complementary to those found when growth trajectories were altered by temperature rather than dietary manipulations, show that the costs of accelerated growth can last well beyond the time over which growth rates differ and are affected by the time available until an approaching life‐history event such as reproduction.

## Introduction

Senescence is a late‐life decline in physiological functioning with age that results in a decrease in organismal performance such as locomotor ability, reproduction or an increase in mortality rate (Williams 1957; Hamilton 1966; Abrams 1991; Rose 1991). The pattern and pace of senescence is thought to be influenced by trade‐offs in the allocation of limiting resources to self‐maintenance and other activities, such as reproduction (called disposable soma theory; Kirkwood 1977). These trade‐offs might produce individual variation in rates of senescence (a major assumption of life‐history theory; Stearns 1992) because individuals are expected to maximize their fitness by allocating energy and resources between current and future reproduction (Schaffer 1974; Partridge 1992), and the precise allocation will vary according to an individual's state (Hendry *et al*. 2004; Bouwhuis *et al*. 2010). It is known that optimal strategies of resource allocation also depend on environmental conditions: the optimal allocation to growth will thus vary over time, depending on resource availability as well as current nutritional state (Mangel & Munch 2005; Lee *et al*. 2011).

Compensatory growth is a well‐known strategic adjustment that occurs when growth rate is accelerated upon refeeding after a period of environmentally suppressed growth; if complete, it results in normal adult size still being attained despite the earlier setback [i.e. full catch‐up growth, following the terminology of Hector & Nakagawa (2012)]. While such growth compensation might carry costs, there may be overall fitness benefits. In populations that experience high rates of juvenile predation, accelerated growth can increase survival by reducing the period spent at a vulnerable size (Arendt 1997; Sogard 1997). Moreover, larger individuals may have greater competitive abilities (Johnston 1993) and an earlier age of maturation (Rowe & Thorpe 1990). Notwithstanding these benefits of compensatory growth, previous work has demonstrated that growth acceleration can also have negative effects in later life. The hyperphagic response needed for increased growth after food restriction could increase the risk of predation while foraging (Ali & Wootton 2000), while the physiological process of growth acceleration may cause increased cellular damage and metabolic costs (Tarry‐Adkins *et al*. 2013) which could, for instance, reduce future life span or reproductive capacity (Metcalfe & Alonso‐Alvarez 2010). It has been reported that compensatory growth in fish induced by earlier food restriction causes a reduced ability to swim against fast‐flowing water (Álvarez & Metcalfe 2005) and a reduced life span (Inness & Metcalfe 2008), as well as negative effects on reproductive output (Auer *et al*. 2010). However, while several studies have documented the existence of a compensatory growth response to a period of poor nutrition during early life (reviewed by Ali, Nicieza & Wootton 2003), there has been surprisingly little effort to study the long‐term effects of this cause of growth acceleration on rates of senescence.

Metcalfe, Bull & Mangel (2002) hypothesized that the amount of time available to catch up after a period of poor growth would influence patterns of compensatory growth: less time available until a key event such as breeding might result in increased pressure for accelerated growth (the so‐called time‐stress hypothesis). However, in these situations, why should animals opt to accelerate their growth as opposed to growing normally and breeding at a smaller size and/or continuing to grow through the breeding season? It has been shown that an increased body size has reproductive benefits for both sexes in terms of mate choice (Howard *et al*. 1998), which is important at the beginning of the breeding season. Moreover, rapid somatic growth prior to the breeding season would allow more time for gonad growth and hence increased fecundity or sperm production. The tendency to show a catch‐up growth response may vary between populations in relation to the likelihood that they would naturally experience a time constraint (Dahl *et al*. 2012; Orizaola, Dahl & Laurila 2014), suggesting that time constraints influence whether the benefits of faster growth would outweigh the costs of growth compensation. Metcalfe & Alonso‐Alvarez (2010) argued that the extent of growth acceleration should be flexible under time stress since reduced time available prior to a life‐history event such as reproduction would affect the ability of the animal to repair any molecular or tissue damage that had occurred as a result of the accelerated growth. Indeed, Álvarez & Metcalfe (2005) found that compensatory growth caused a greater decrease in swimming performance in three‐spined sticklebacks (*Gasterosteus aculeatus*) when this occurred close to the breeding season; our recent work using temperature manipulations to alter growth patterns in the same species also showed that this cost was increased when the fish perceived a greater time stress due to photoperiod manipulation that decreased the time available for growth prior to breeding (Lee, Monaghan & Metcalfe 2010, 2012, 2013).

Dietary manipulations can be used to alter growth trajectories, with a brief period of food restriction often inducing compensatory growth once animals are returned to their previous food levels (e.g. Álvarez & Metcalfe 2005; Inness & Metcalfe 2008). The degree of time stress can be altered by using shifted photoperiod regimes that alter the perceived time of year (e.g. time until a key event such as the breeding season). By using both dietary and photoperiod manipulations to manipulate growth rates in three‐spined sticklebacks at two different times of year, we examined: (i) whether compensatory growth in early life alters the subsequent pattern of age‐related changes in two key traits related to adult fitness (swimming endurance and breeding ornamentation), (ii) how changes in these traits with age relate to life span, and (iii) how these patterns are affected by the degree of time stress. While the negative effects of compensatory growth in sticklebacks have already been documented for some life‐history traits, our approach here is to investigate these questions over longer time periods so as to produce a more complete analysis of the effects of compensatory growth on life histories. Our hypotheses were that compensatory growth would induce faster rates of decline in locomotor and reproductive traits (i.e. a faster rate of senescence in performance traits), that these trends would be associated with changes in life span and that effects would be greater in fish perceiving greater time stress.

## Materials and methods

### Fish and rearing conditions

We presumed that the amount of time available for growth prior to the onset of the breeding season might affect the compensatory growth response, so the same experimental treatments were conducted at two time points with independent fish (hereafter referred to as the Winter and Spring experiments; experimental design shown in Fig. S1a). On 1 November 2007 (= Winter experiment) and 29 January 2008 (= Spring experiment), wild juvenile three‐spined sticklebacks (*Gasterosteus aculeatus*) were captured with a dip net and minnow traps in the River Endrick, Scotland, UK (56°04′N, 4°23′W). All fish were transferred to acclimatization aquaria (80 L and density 2 fish L^–1^) for 3 weeks and fed *ad libitum* frozen chironomid larvae. Prior to the start of experiments, the temperature was maintained at 9·7 ± 0·1 °C, while the photoperiod was ambient. All fish were anaesthetized and measured for standard length (±0·01 mm) and wet mass (±0·001 g) on 21 November 2007 and 21 February 2008 for the Winter and Spring experiments, respectively. We then sorted fish into groups of five (of differing size, to aid identification), with each group of five fish in a separate tank (335 × 170 × 185 mm) provided with aeration, a filter and artificial plants as well as an additional 62·5 mL of seawater per tank to prevent the risk of whitespot infection (*Ichthyophthirius multifiliis*).

### Dietary and time‐stress manipulation

**Figure 1:**
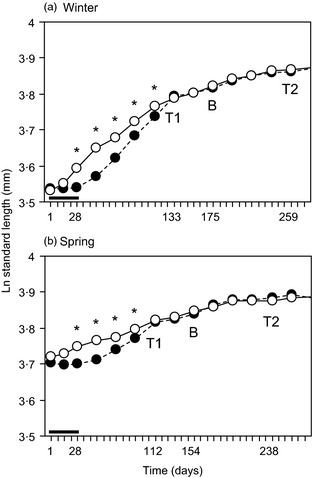
Growth trajectories (logarithm of standard length in mm) of three‐spined sticklebacks (*Gasterosteus aculeatus*) over the early compensatory period in the (a) Winter and (b) Spring experiment. Note that the two experiments started on different days, so that day 1 is 21 November 2007 in (a) and 21 February 2008 in (b). The thick horizontal line indicates the period of dietary manipulation (28 days); black circles and dashed line represent the restricted diet and open circles and solid line the *ad libitum* control. Asterisks indicate significant differences in length between treatment groups (*P *<* *0·05). ‘T1’ and ‘T2’ indicate the timing of swimming trials (i.e. at the end of the period of compensatory growth and 18 weeks later, after the breeding season). The temperature for both groups was kept at 10 °C until the start of the first breeding season (‘B’), at which point the temperature was raised to 14 °C and male sticklebacks were isolated from female sticklebacks (see Materials and methods for more details).

Four replicate tanks of five fish were assigned randomly to each manipulation, defined in relation to dietary regime: restricted (R group, fed 2% of body mass) and control (C group, fed *ad libitum*) (Fig. 1a). The control group are the same fish as used as controls in temperature manipulation experiments in Lee, Monaghan & Metcalfe (2010, 2012, 2013), and so data from these fish are used simply as a comparison with the novel data from the food restricted group. A diet of 2% of body mass was chosen for the growth suppression period since this has earlier been found to produce reduced growth at 10 °C (Allen & Wootton 1984). At the end of a 4‐week manipulation period (Period 1), all fish were returned to an *ad libitum* diet for the rest of the experiment (Period 2; Fig. S1b; Table S1). The temperature was held at 10 °C during periods 1 (manipulation) and 2 (compensation), but was raised to 14 °C during each breeding season in line with ambient temperature. The first and second breeding seasons were defined as periods 3 and 5, respectively, with Period 4 being the intervening non‐breeding season; full details of the time periods are given in the supplementary information (Appendix S1).

The fish in the Spring experiment had less time available to recover from the growth perturbation compared to those in the Winter experiment, allowing us to examine the time‐stress hypothesis. As an additional manipulation of time stress, we also gave fish in each experiment an ambient photoperiod regime (AP) or a day length which corresponded to a point 35 days earlier in the season [= delayed photoperiod (DP)], thereby delaying the perceived time to the onset of breeding in DP fish (alleviating time stress). Fluorescent lights were controlled by electronic timers, and the lighting regimes were manipulated using blackout plastic sheeting around the tanks in the delayed groups. Both diet and photoperiod were manipulated in a factorial design, yielding four manipulation groups in each experiment (each with four replicate tanks). Since the control groups (= normal diet ration) experienced *ad lib* food at a constant 10 °C during periods 1 and 2, they showed steady growth up until the breeding season (Lee, Monaghan & Metcalfe 2010); the restricted diet groups (= fed 2% of body mass for a 4‐week period, followed by *ad lib* food) were expected to show slowed growth followed by (compensatory) growth acceleration. Following results obtained for a parallel perturbation of growth trajectories using temperature manipulations (Lee, Monaghan & Metcalfe 2010), fish in the Winter experiment (being under reduced time stress) were predicted to show weaker compensatory responses than those in the Spring experiment, while if fish were sensitive to photoperiod, then fish in the delayed groups within each experiment should show weaker compensatory responses (due to reduced time stress) than their corresponding group exposed to a normal photoperiod.

We remeasured the fish for length and mass every 2 weeks during the dietary manipulation period and every 3 weeks thereafter; all fish were starved for 24 h prior to measuring to prevent variation in the weight of stomach contents. Tanks were inspected daily in order to monitor mortality rates throughout the experiment.

### Quantification of swimming performance and sexual ornamentation

The swimming performance of the fish was quantified as the amount of time a fish could swim against a constant current of water known to be too strong for sustained swimming, a measure of swimming stamina used in previous studies (Ojanguren & Braña 2000, 2003; Álvarez & Metcalfe 2005; Royle, Metcalfe & Lindstrom 2006). The full details of the experimental set‐up are given elsewhere (see Álvarez & Metcalfe 2005; Lee, Monaghan & Metcalfe 2010) and in the electronic supplement (Appendix S1). In both experiments, we measured swimming performance twice (‘T1’ and ‘T2’ in Fig. S1b), first when fish in the different manipulation groups had finished the phase of compensatory growth and had converged on the same mean size prior to breeding, and again 18 weeks later (at the end of the first breeding season). Swimming endurance is defined as the amount of time that a fish swam at the highest flow rate.

At the onset of the breeding season, the colour of the eye sclera in both male and female sticklebacks changes from silver to blue or blue‐green (Barber *et al*. 2000) and the intensity of the eye colour is positively correlated with mating condition (Kraak, Bakker & Mundwiler 1999; Flamarique *et al*. 2013), so eye colour can be used as a readily quantifiable measure of breeding ornamentation that can be applied to both sexes. At the beginning of each breeding season (periods 3 and 5), we allocated each male that was developing the characteristic breeding coloration of blue eyes and a red throat to an individual tank, which was of the same size and arrangement as its group tank (this breeding experimental protocol is illustrated in the electronic supplementary material, Appendix S1). As the intensity of blue eye coloration in this population tends to remain high for about 4 months over the breeding season and then declines sharply, we began weekly measurements of eye coloration at the end of the dietary manipulation (25 December 2007 for the Winter experiment and 25 March 2008 for the Spring) until the end of the second breeding season using a 4‐point scale (Boughman 2007; Lee, Monaghan & Metcalfe 2012): 0 (no blue coloration) to 4 (strong bright blue coloration). All fish were scored by the same person using this standardized procedure on the same day. The duration of breeding ornamentation was then defined as the number of weeks during each breeding season that fish maintained a relatively strong blue eye colour (3 + for males, 2 + for females). Note that while female fecundity and male red throat coloration and rate of nest building were also quantified (see supplement information; Appendices S1 and S2; Tables S2‐S5; Figs S3‐S6), these data are not considered in the main analyses since relatively small sample sizes of fish surviving to the second breeding season (and hence allowing calculation of changes over successive breeding seasons) made it important to focus on traits where the data set could include both sexes.

The change in traits with age was defined as follows: *change* = ((*α*_1_ + *α*_2_)/*α*_1_) – 2, where *α*_1_ is the earlier measurement and *α*_2_ the later one (for swimming endurance, the measurements were at the beginning and end of the first breeding season; for breeding ornamentation, the values were the duration above the threshold score in the first and second breeding seasons). Values greater than zero thus indicate an increase (and values less than zero a decrease) in the trait value over time.

### Compensatory growth rate

Compensatory growth rate (% per day) was defined as the growth rate during the compensatory period (Period 2) after the dietary manipulation and was calculated as follows: *compensatory growth rate* = 100·[*ln*(*L*_*c*_ ·*L*_*i*_^−1^)]·*t* ^−1^, where *L*_*i*_ was the length at the end of Period 1 and *L*_*c*_ was the standard length when fish in the different manipulation groups had finished the phase of compensatory growth and had appeared to converge on the same mean size prior to breeding (based on inspection of growth trajectories), and *t* was the interval in days between *L*_*c*_ and *L*_*i*_, being 105 days in the Winter experiment and 84 days in the Spring experiment.

### Statistical analysis

To test for differences in body length and mass between treatment groups, multivariate analysis of variance (manova) was used at the beginning of each experiment before fish had been allocated to their treatment tanks as well as at the end of the compensatory period. We used linear mixed‐effect models (LMEs) in order to analyse the effects of the dietary and photoperiod manipulations on compensatory growth rate in both experiments, with season of experiment (Winter or Spring), dietary (restricted or control, denoted R and C, respectively) and photoperiod (AP or DP) treatments and sex (male or female) as fixed effects, fish length (manipulated length at the end of Period 1) as a covariate and tank as a random effect, plus all interactions.

Any association between compensatory growth rate and other traits could be caused by an effect of dietary treatment (i.e. a between‐treatment group effect) or an effect due to variation in individuals within a dietary treatment group (i.e. a within‐treatment group effect). To distinguish within‐ from between‐group effects, we used the technique of ‘within‐group centring’ (van de Pol & Wright 2009). A first variable that expresses only the between‐group variance component was given by the mean value of the trait in question, calculated over all fish in the same dietary treatment group. A second variable that expresses only the within‐group variance component was computed by subtracting this treatment mean value from the individual values in a given dietary treatment group. LME based on within‐group centring was used to analyse the factors influencing age‐related changes in swimming endurance and in the duration of breeding ornamentation. The full model included season of experiment, photoperiod treatment and sex as fixed effects and mean compensatory growth rate for a dietary treatment (as a between‐group effect) and within‐group variation about this treatment mean for compensatory growth rate (as the within‐group effect) as covariates. We also included the identity of the original rearing tank as a random effect, plus all 2‐way interactions. Equivalent LMEs subsequently explored the factors influencing life span; the full models contained experiment, photoperiod treatment and sex as fixed effects and compensatory growth rate (partitioned into within‐ and between‐dietary treatment effects as above), change in swimming endurance and change in the duration of breeding ornamentation (similarly partitioned into within‐ and between‐dietary treatment effects) as covariates, all 2‐way interactions, and the random effect of rearing tank identity.

For all models, we started with a full model and sequentially dropped non‐significant variables so that the final models only included significant terms (or terms that were components of significant interactions). All means are presented with standard errors, and all of the analyses were performed with the software *R* v.2.15.2 (R Development Core Team 2012) and the package *lme4* (Bates, Maechler & Bolker 2012). All experiments were performed under licence from the UK Home Office (PIL 60/11377).

## Results

### Compensatory growth rate

**Figure 2:**
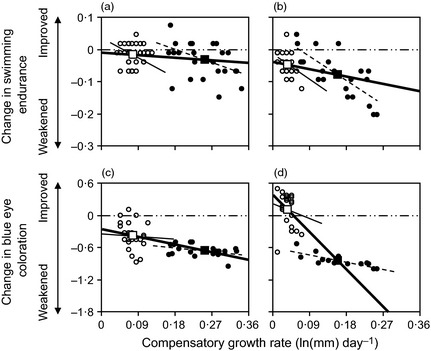
Age‐related changes in swimming endurance (a and b) and duration of breeding ornamentation (c and d) of three‐spined sticklebacks in relation to their earlier rate of compensatory growth. Zero change is indicated by the double‐dashed line. Individual data points and within‐treatment regression lines are plotted from the Winter (left panels) and Spring (right panels) experiments, categorized by dietary treatment (restricted: black circle and dashed line; control: open circle and thin solid line). Larger square symbols and thicker solid lines denote treatment mean values and between‐treatment regression lines (see van de Pol & Wright 2009 for statistical explanation and Table 2 for full statistical analysis).

At the beginning of the two experiments, there was no difference in the mean standard length or mass of sticklebacks allocated to the dietary treatment groups (manova, Winter: Wilks’ *λ* = 0·987, *F*_2,77_ = 0·52, *P *=* *0·132; Spring: Wilks’ *λ* = 0·988, *F*_2,77_ = 0·46, *P *=* *0·636) or the two photoperiod treatments (Winter: Wilks’ λ = 0·999, *F*_2,77_ = 0·03, *P *=* *0·975; Spring: Wilks’ *λ* = 0·999, *F*_2,77_ = 0·01, *P *= 0·995). In the Winter experiment, the mean standard length of R fish at the end of the manipulation period (= manipulated length) was significantly smaller than that of C fish (linear mixed‐effect models (LMEs), *F*_1,12·69_ = 6·08, *P *=* *0·029; Fig. 2a), but there was no effect of photoperiod treatment (*F*_1,11·68_ = 0·06, *P *=* *0·806) or sex (*F*_1,49·36_ = 0·41, *P *=* *0·523). Not surprisingly, there was a positive effect of initial length at the start of the Winter experiment on the fish's length at the end of the manipulation period (*F*_1,46·59_ = 123·89, *P *<* *0·001). The analysis of the Spring experiment found similar effects of initial length (*F*_1,64·36_ = 5238·98, *P *<* *0·001) and dietary treatment group (*F*_1,12·95_ = 108·73, *P *<* *0·001; Fig. 2b) on manipulated length, but also a significant effect of photoperiod treatment (*F*_1,12·94_ = 6·76, *P *=* *0·022), with fish being larger at the end of the manipulation period under the ambient than under the delayed photoperiod. Again there was no effect of sex (*F*_1,67·09_ = 0·42, *P *=* *0·521). Broadly similar results were obtained for analyses of fish body mass in both experiments (see Appendix S2).

When again given food *ad libitum*, R fish grew rapidly so that after 15 weeks in the Winter experiment and 12 weeks in the Spring experiment, the differences in length and mass between R and C fish were no longer significant (Winter: Wilks’ *λ* = 0·937, *F*_2,64_ = 2·16, *P *=* *0·123, Spring: Wilks’ *λ* = 0·978, *F*_2,69_ = 0·79, *P *=* *0·458), nor were there differences between photoperiod groups (Winter: Wilks’ λ = 0·996, *F*_2,64_ = 0·12, *P *=* *0·885, Spring: Wilks’ *λ* = 0·968, *F*_2,69_ = 1·14, *P *=* *0·325). Compensatory growth rates (= growth rate during the compensatory period, Period 2) were significantly higher in the Winter experiment than in the Spring experiment (Table 1 and Fig. 1). While there was no effect of photoperiod (LME, *F*_1, 97·8_ = 0·15, *P *=* *0·700) or sex (*F*_1, 97·8_ = 0·14, *P *=* *0·709) on compensatory growth rate, it was affected by dietary treatment and manipulated length, with the growth of R fish and of smaller fish being greatest (Table 1, Fig. 1). There was also a significant interaction between season and dietary treatment (Table 1), the fastest growth rate being that of R fish in the Winter experiment (Fig. 1).

**Table 1:** Compensatory growth rate in relation to dietary and photoperiod treatments in the Winter and Spring experiments. The full linear mixed‐effect model (LME) included season of experiment (Winter or Spring), dietary (restricted or control) and photoperiod (ambient or delayed) treatments as fixed effects, manipulated fish length (at the end of Period 1) as a covariate and tank as a random effect, plus interactions among variables. Non‐significant variables were dropped from the final model. Note that a positive estimate is associated with a faster rate of compensatory growth. The parentheses represent the reference coding of the categorical variable

Final model	Estimate ± SE	*F*	d.f.	*P*
Intercept	0·600 ± 0·098			
Season (Winter)	0·065 ± 0·014	14·80	1, 98·80	<0·001
Dietary (Restricted)	0·164 ± 0·011	288·54	1, 98·80	<0·001
Manipulated fish length	−0·102 ± 0·029	12·59	1, 98·80	<0·001
Season (Winter) × dietary (Restricted)	0·046 ± 0·016	8·44	1, 98·80	0·004

### Effect of growth trajectories on swimming endurance

Swimming endurance prior to the first breeding season was not significantly different between fish from the Winter and Spring experiments (LME, *F*_1, 25·23_ = 0·93, *P *=* *0·343) nor between photoperiod treatment groups (*F*_1, 23·52_ = 3·68, *P *=* *0·067). The endurance of R fish in this first test, however, was significantly lower than that of C fish (*F*_1, 25·22_ = 18·07, *P *<* *0·001). There was also an effect of fish length at the first test (*F*_1, 118·89_ = 123·36, *P *<* *0·001): the larger the fish's length at the end of the dietary manipulation period, the greater its swimming endurance (see Fig. S2).

The declines in swimming endurance over the first breeding season were greater in the Spring than in the Winter experiment (Fig. 2a,b), and were significantly affected by both between‐ and within‐dietary treatment effects of compensatory growth (Table 2): the greater the rate of compensatory growth in the period leading up to the breeding season, the greater the decline in endurance. While there was no effect of sex on the change in endurance (*F*_1, 96·55_ = 0·039, *P *=* *0·845), it was influenced by photoperiod, with the delayed fish showing less deterioration in swimming endurance than did the ambient fish (Table 2). The interaction between experiment and within‐treatment variation of compensatory growth affected this change (Table 2), since a given rate of growth led to a bigger decline in swimming endurance in the Spring than in the Winter experiment (Fig. 2a,b).

**Table 2:** Linear mixed‐effect model (LME) analyses of factors predicting changes in swimming endurance and in the duration of breeding ornamentation (blue eye colour) of three‐spined sticklebacks. In both cases, the full models included season of experiment (Winter or Spring), photoperiod treatment (ambient or delayed) and sex (male or female) as fixed effects, compensatory growth rate (partitioned into between‐group effects due to diet treatment, and within‐group effects due to remaining individual variation) as a covariate, and tank as a random effect, plus 2‐way interactions. Non‐significant variables were dropped from the final models. Note that for swimming endurance a positive estimate indicates an increase in endurance over the breeding season, while for breeding ornamentation it indicates a longer period of blue eye coloration in the second than in the first breeding season. The parentheses represent the reference coding of the categorical variable

Analysis	Final model	Estimate ± SE	*F*	d.f.	*P*
Swimming endurance	Intercept	−0·037 ± 0·010			
	Season (Winter)	0·047 ± 0·010	20·50	1, 15·68	<0·001
	Photoperiod (Ambient)	−0·021 ± 0·010	4·94	1, 16·60	0·041
	Between‐group effect of growth	−0·148 ± 0·062	5·59	1, 15·86	0·031
	Within‐group effect of growth	−0·829 ± 0·143	26·56	1, 93·44	<0·001
	Season (Winter) × within‐group effect of growth	0·626 ± 0·200	9·75	1, 93·61	0·002
Breeding ornamentation	Intercept	0·488 ± 0·060			
	Season (Winter)	−0·606 ± 0·082	54·74	1, 19·01	<0·001
	Sex (Male)	−0·321 ± 0·081	15·88	1, 76·63	<0·001
	Between‐group effect of growth	−8·174 ± 0·558	188·16	1, 31·90	<0·001
	Within‐group effect of growth	−1·561 ± 0·545	8·22	1, 76·56	0·005
	Season (Winter) × between‐group effect of growth	5·956 ± 0·636	87·76	1, 28·25	<0·001
	Sex (Male) × between‐group effect of growth	1·256 ± 0·542	5·37	1, 76·26	0·023

### Effect of growth trajectories on the duration of breeding ornamentation

The period over which fish maintained a blue eye colour above the threshold level in the first breeding season (= 2008) was significantly longer in the Winter than in the Spring experiment (LME, *F*_1, 24·26_ = 22·11, *P *<* *0·001) and longer in males than in females (*F*_1,77·91_ = 47·46, *P *< 0·001). While there were no effects of dietary treatment (*F*_1,23·75_ = 1·91, *P *=* *0·180) or photoperiod (*F*_1,23,39_ = 0·11, *P *=* *0·746) on this duration, there was a significant interaction between season of experiment and dietary treatment (*F*_1,23·02_ = 16·74, *P *<* *0·001): C fish in the Winter experiment maintained their coloration for the longest duration, while R fish in the Spring were the shortest.

The reduction in the duration of breeding coloration between the first (= 2008) and second (= 2009) breeding seasons was greater in the Spring than in the Winter experiment, and greater in males than in females (Table 2). The age‐related change in breeding ornamentation was unaffected by photoperiod treatment (*F*_1, 28·28_ = 0·069, *P *=* *0·795) but was significantly affected by compensatory growth rate, with faster growth (both between dietary treatments and at an individual level within a treatment) being associated with a bigger reduction in eye ornamentation in the second breeding season (Table 2). The significant interactions (Table 2) indicated that the deleterious effect of growth rate on the decline in breeding ornamentation was greater in the Spring experiment (Fig. 1) and in females.

### Life span

The exposure to different dietary rations during Period 1 had no immediate or direct effect on mortality patterns because no fish died during the period of dietary manipulation. Most fish (83·1%) were still alive at the beginning of the first breeding season (a typical survival rate for juvenile fish under laboratory conditions), with no evident differences in pre‐breeding survival between the treatment groups. However, survival decreased from the first breeding season onwards, so that only 57·5% of fish were alive by the beginning of the second breeding season.

Life span was significantly affected by the season of experiment (i.e. Winter vs. Spring; Table 3). On average, fish in the Spring experiment, which had been under greater time stress, died at a younger age than did those in the Winter experiment (the median life span of fish in the Spring experiment was 735 days, whereas that of fish in the Winter experiment was 802 days). This is despite the Spring experiment not having started until the fish were almost adult and so only including fish that had already passed the juvenile stage naturally associated with relatively higher mortality. There was also a significant effect of sex on life span (Table 3), with males dying sooner than females in both experiments (Winter: median life span = 730 days in males, 966 days in females; Spring: 557 days in males, 841 days in females). However, life span was not affected by photoperiod (*F*_1,24·60_ = 2·81, *P *=* *0·106), nor was it related to the rate of compensatory growth (between‐group effect of growth, *F*_1,21·10_ = 3·38, *P *=* *0·080; within‐group effect of growth, *F*_1,74·15_ = 0·707, *P *=* *0·403).

**Table 3:** Life span of three‐spined sticklebacks in relation to changes in their swimming endurance and in the duration of breeding ornamentation (blue eye colour). The full model included season of experiment (Winter or Spring), photoperiod treatment (ambient or delayed) and sex (male or female) as fixed effects, compensatory growth rate, age‐related changes in swimming endurance and in the duration of breeding ornamentation (partitioned into between‐group effects due to diet treatment, and within‐group effects due to remaining individual variation) as covariates, and tank as a random effect, plus 2‐way interactions. Non‐significant variables were dropped from the final model. Note that a positive estimate indicates a longer life span. The parentheses represent the reference coding of the categorical variable

Final model	Estimate ± SE	*F*	d.f.	*P*
Intercept	971·727 ± 14·236			
Season (Winter)	67·443 ± 17·261	15·27	1, 24·72	0·001
Sex (Male)	−34·707 ± 17·336	4·01	1, 77·49	0·049
Within‐group effect of swimming endurance	569·979 ± 167·019	11·65	1, 77·28	0·001
Between‐group effect of breeding ornamentation	101·304 ± 24·049	17·74	1, 24·42	<0·001
Within‐group effect of breeding ornamentation	185·904 ± 38·039	23·89	1, 77·68	<0·001

**Figure 3:**
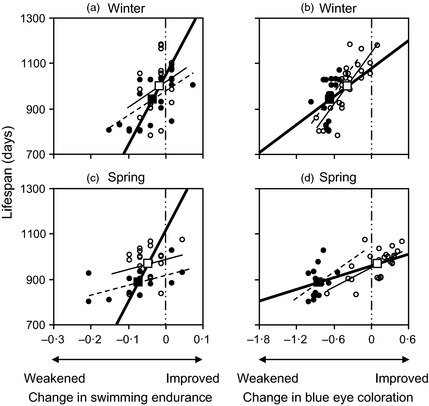
Life span in three‐spined sticklebacks in relation to age‐related changes (square root transformed) of swimming endurance (*a* and *c*) and blue eye coloration (*b* and *d*). Zero change is indicated by the vertical double‐dashed line. Individual data points and within‐treatment regression lines are plotted from the Winter (*a* and *b*) and Spring (*c* and *d*) experiments, categorized by dietary treatment (restricted: black circle and dashed line; control: open circle and thin solid line). Larger square symbols and thicker solid lines denote treatment mean values and between‐treatment regression lines (see van de Pol & Wright 2009 for statistical explanation and Table 3 for full statistical analysis).

Life span was predicted by the change over time in the two measures of whole‐organism performance. While there was no between‐group effect of the change in swimming endurance (*F*_1,21·34_ = 3·38, *P *=* *0·080), there was a significant within‐group effect (Table 3): the faster the deterioration in swimming endurance over the first breeding season, the shorter was the fish's life span (Fig. 3a,c). Similarly, a greater reduction in the duration of breeding ornamentation between the first and second breeding season was associated with a shorter life span, with significant between‐ and within‐treatment group effects (Table 3; Fig. 3b,d).

## Discussion

We have investigated how compensatory growth induced by changes in food availability in early life affected age‐related declines in swimming endurance and in the duration of sexual ornamentation, as well as how these rates of senescence in organismal traits are associated with life span. A compensatory (i.e. accelerated) growth trajectory was successfully induced by restricting the availability of food during a short‐term period in juvenile life. As predicted, growth acceleration caused a more rapid deterioration in both locomotor endurance and breeding ornamentation, and a shorter life span. Moreover, these long‐term negative effects of a compensatory growth trajectory were more pronounced when a reduced amount of time (either to catch up in size or to recover from that growth acceleration) was available before the commencement of the breeding season, supporting the predictions of the time‐stress hypothesis (Metcalfe, Bull & Mangel 2002).

Unexpectedly, growth rates during the phase of compensatory growth were on average faster in the Winter experiment (despite the fish being under less time stress than in the Spring experiment), but this may have been because fish in the Winter experiment were younger and smaller in body length at the beginning of the experiment than in the Spring experiment. In general, however, the subsequent performance (in terms of both senescence in locomotion and reproduction) and longevity of the R fish were less affected in the Winter than in the Spring experiment, despite the faster compensatory growth of this group of fish. This may have been because of the greater time available for fish to recover from any damage caused by fast growth in the Winter experiment – a similar result for swimming performance was found by Álvarez & Metcalfe (2005) and Lee, Monaghan & Metcalfe (2010).

It is clear that growth and development in animals may incur significant costs (Roff 2002). The fish were able to accelerate their growth after the period of food restriction presumably through hyperphagia (Ali & Wootton 2000), but it is well known that accelerated growth negatively affects the development of muscle cellularity (Galloway, Kjorsvik & Kryvi 1999; Johnston *et al*. 2002). Álvarez & Metcalfe (2005) showed that swimming endurance was lower in fish that had previously been subjected to food restriction and had then gone through a phase of compensatory growth, possibly due to changes in cellular structure caused by the accelerated growth. Compensatory growth may lead to cumulative increases in oxidative damage to biomolecules due to greater production of reactive oxygen species or decreased investment in antioxidant protection or repair (Samuels & Baracos 1995; Tarry‐Adkins *et al*. 2013). This oxidative damage is a strong candidate for senescence‐related changes in individuals (Stadtman 1992; Hamilton *et al*. 2001) because oxidative damage can be correlated with a decline in physiological functioning with age (= senescence). Therefore, the reduction in physical endurance over the breeding season (locomotor senescence) and the reduction in sexual ornamentation with age, especially in fish that had undergone accelerated growth, may be a consequence of increased levels of accumulated biomolecular damage, further exacerbated by the cost of breeding. While the mechanisms underlying the links between early growth rate, senescence and life span are currently not known, a link to oxidative stress seems plausible since studies have suggested that increased oxidative stress levels and rates of cellular damage and senescence induced by compensatory growth may accelerate telomere shortening (Jennings, Ozanne & Hales 2000; Tarry‐Adkins *et al*. 2008; Geiger *et al*. 2012), which itself is linked to life span (Heidinger *et al*. 2012; Boonekamp *et al*. 2014).

Under conditions of finite resources, natural selection favours allocation strategies that will maximize long‐term fitness over the organism's life span. It is well documented that reproductive effort and investment are affected by a trade‐off between growth and reproduction (Stearns 1989; Green & Kaker 1991). For instance, Poizat, Rosecchi & Crivelli (1999) showed that female sticklebacks lose somatic condition over the course of the breeding season but increase their investment in gonad weight relative to body weight. Presumably, a phase of restricted food availability in early life may affect this later resource allocation between soma and gonads; while compensatory growth may partly be achieved through hyperphagia (Ali & Wootton 2000), as was predicted in a related exercise modelling optimal patterns of growth following a period of growth retardation (i.e. faster rate of food consumption during the phase of compensatory growth, Lee *et al*. 2011), this may not be sufficient to achieve the desired growth rate, leading to changes in resource allocation. A diversion of resources towards skeletal growth could negatively affect the development of reproductive tissues, in a similar manner to the way in which it is thought to interfere with the development of non‐reproductive structures (Ricklefs, Shea & Choi 1994; Arendt, Wilson & Stark 2001; Arendt 2003). The accumulation of damage can also negatively affect reproductive investment. There is increasing evidence of a negative relationship between oxidative stress and reproductive capacity in wild organisms (Bize *et al*. 2008; Perez, Lores & Velando 2008), suggesting that oxidative stress may in some way constrain reproduction (Metcalfe & Alonso‐Alvarez 2010). There are thus different mechanisms through which compensatory growth might result in accelerated reproductive and whole‐organism senescence and reduced life span, but further experiments that include measurements of oxidative damage and repair rates are needed to distinguish between these hypotheses.

Growth and reproduction in ectotherms are sensitive to both temperature and photoperiod; while both of these environmental factors can indicate the time of year, photoperiod is thought to be the cue used most often as a time reference since it is not susceptible to temporal fluctuations. The photoperiod manipulations in general had less of an effect than the direct manipulation of time available (i.e. the comparison of the Winter and Spring treatments), but nonetheless had an effect on locomotor performance, showing that a shift in the perceived time of year affected the extent of the negative impact of compensatory growth. This supports the ‘time‐stress’ hypothesis that both growth rate and resource allocation decisions can be affected by the perception of time of year and, in particular, the time available until key life‐history events (Metcalfe, Bull & Mangel 2002). The observed pattern for the negative effect of a given growth rate to be more pronounced when the perceived time stress was shorter (i.e. both in the Spring experiment and under the ambient rather than delayed photoperiod) may be due to changes in the trade‐off between growth and reproductive investment, since when time was short there was less time to repair any damage incurred by growth acceleration. In other words, an increased time stress might induce more resources to be allocated to growth (with less to reproduction), so altering the effects of compensatory growth on subsequent swimming endurance and reproductive investment. The effects of time stress on growth rates have been found in a range of taxa (Gotthard 2008). Time conflicts between growth rate and reproduction can also occur after the reproductive season (Dawson *et al*. 2000). Therefore, we suggest that the degree of time stress interacts with prior growth trajectory to determine the animal's optimal current rate of growth, taking into account the trade‐off between growth and reproduction and the effect of accelerated growth on performance in later life. However, this hypothesis requires more investigation.

In summary, while compensatory growth led to individuals catching up in size after a period of poor food rations, and so may have had a short‐term beneficial impact on reproductive success, this study showed significant negative effects of such accelerated growth on both locomotor (swimming) endurance and the degree of reproductive investment. The negative effects became stronger rather than weaker over time (e.g. with faster growth leading to faster declines in swimming performance over the breeding season and a reduced investment in reproduction in the second year). Moreover, the perception of amount of time available prior to breeding altered these costs of compensatory growth. Future studies are needed to determine the mechanisms underlying these effects.

## Supporting information


**Lay Summary**

**Appendix S1.** Additional methods.
**Appendix S2.** Additional results.
**Table S1.** Description of experimental manipulations. Note that during Period 1 Restricted (R) fish were fed a restricted diet (2% of body mass) while Control (C) fish were fed *ad libitum*.
**Table S2.** Mixed model analyses of red throat colouration of male sticklebacks in relation to age (first breeding or second breeding), season of experiment (Winter or Spring), dietary (restricted or control) and photoperiod (ambient or delayed) treatments, manipulated fish length (at the end of the dietary manipulation, ln transformed) and compensatory growth rate after the 4 weeks of dietary manipulation, plus tank as a random effect.
**Table S3.** Mixed model analyses of time required by male sticklebacks to build a nest in relation to age (first breeding or second breeding), season of experiment (Winter or Spring), dietary (restricted or control) and photoperiod (ambient or delayed) treatments, manipulated fish length (at the end of the dietary manipulation, ln transformed) and compensatory growth rate after the 4 weeks of dietary manipulation, plus tank as a random effect.
**Table S4.** No. of eggs in 1st clutch and mean mass of an egg from that clutch in relation to season of experiment (Winter or Spring), dietary (restricted or control) and photoperiod (ambient or delayed) treatment, length at the time of spawning (ln transformed) and compensatory growth rate after the 4 weeks of dietary manipulation in the Winter and Spring experiments.
**Table S5.** Proportion that the eggs produced in the first breeding season made up of the total number of eggs produced by a female over both the first and second breeding seasons, in relation to season of experiment (Winter or Spring), dietary (restricted or control), photoperiod (ambient or delayed), length at time of spawning (ln transformed) and compensatory growth after the 4 weeks of dietary manipulation in the Winter and Spring experiments.
**Table S6.** Maximum lifespan (defined as the age by which 90% of the population in a treatment group had died) in relation to dietary and photoperiod treatments.
**Fig. S1.** (a) Illustration of the experimental design, with three treatments (dietary, photoperiod and season of experiment).
**Fig. S2.** Effects of dietary treatment on swimming performance in three‐spined sticklebacks: (a and b) swimming endurance (ln(s)) at the end of the compensatory period in relation to fish length at the time (ln(mm)) and (c and d) change in swimming endurance over the breeding season (as the amount of the advance in the second trial compared to the first trial, see Materials and Methods for a formula) in relation to fish length at time of first swimming test.
**Fig. S3.** No. of weeks that male three‐spined sticklebacks maintained a strong red throat colour (i.e. exceeding the population mean score) in their first (white bar) and second (grey bar) breeding season, in relation to dietary manipulation (restricted or control) and photoperiod regime ((a) ambient or (b) delayed) in both the Winter (left panel) and Spring (right panel) experiments.
**Fig. S4.** Time taken by male three‐spined sticklebacks to build a nest (days, mean ± SE) in relation to dietary manipulation (restricted or control) and photoperiod manipulation ((a) ambient or (b) delayed) in both the Winter (left panel) and Spring (right panel) experiments.
**Fig. S5.** Mean mass of individual eggs (mg, a and c) from the first clutch and size of the first clutch (number of eggs, b and d) produced by one year old female three‐spined sticklebacks during the first breeding period in relation to their length at the time of spawning (mm, ln transformed).
**Fig. S6.** Proportion that the eggs produced in the first breeding season made up of the total number of eggs produced by a female over both the first and second breeding seasons.
