# Supplementary material for: Perturbations in growth trajectory due to early diet affect age‐related deterioration in performance
Source: Funct Ecol. 2015 Aug 29;30(4):625–35. doi: 10.1111/1365-2435.12538 (PMC4994260; doi:10.1111/1365-2435.12538)
Supplement: Supplementary file 1 — Lay Summary [file FEC-30-625-s001.pdf]

Functional Ecology

British Ecological Society

Perturbations in growth trajectory due to early diet affect age-related deterioration in performance

Who-Seung Lee, Pat Monaghan and Neil B. Metcalfe

Dietary conditions affect the growth rates of organisms, which in turn influences future survival and reproduction. Animals can often compensate for a period of slow growth early in life by accelerating their growth when conditions improve, resulting in normal adult size, but this rapid growth can prove costly in later life. In this study we investigated whether this compensatory growth has long-term consequences for patterns of senescence. Using a small species of fish, the three-spined stickleback *(Gasterosteus aculeatus),* we showed that a brief period of reduced food availability in early life affected skeletal growth rate not only during the manipulation itself, but also during a subsequent compensatory phase when fish caught up in size with controls. However, fish that had gone through this period of growth acceleration had a faster decline in their swimming performance and a shorter period of breeding over the following two breeding seasons, suggesting that they were ageing faster. This was confirmed by their having a shorter lifespan. The effects were strongest if the acceleration of growth happened just before the breeding season. These results, complementary to those found when growth trajectories were altered by temperature rather than dietary manipulations, show that the costs of accelerated growth can last well beyond the time over which growth rates differ.

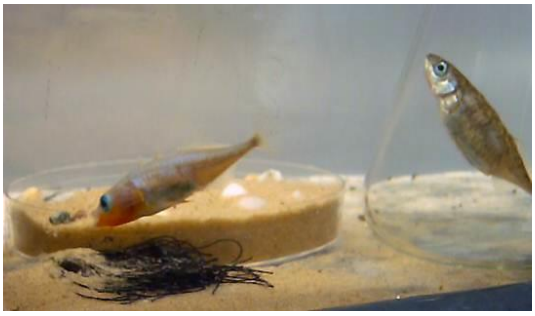

Male (left) and female (right) three-spined sticklebacks
