# Supplementary material for: Perturbations in growth trajectory due to early diet affect age‐related deterioration in performance
Source: Funct Ecol. 2015 Aug 29;30(4):625–35. doi: 10.1111/1365-2435.12538 (PMC4994260; doi:10.1111/1365-2435.12538)
Supplement: Supplementary file 2 — Appendix S1. Additional methods. Appendix S2. Additional results. Table S1. Description of experimental manipulations. Note that during Period 1 Restricted (R) fish were fed a restricted diet (2% of body mass) while Control (C) fish were fed ad libitum. Table S2. Mixed model analyses of red throat colouration of male sticklebacks in relation to age (first breeding or second breeding), season of experiment (Winter or Spring), dietary (restricted or control) and photoperiod (ambient or delayed) treatments, manipulated fish length (at the end of the dietary manipulation, ln transformed) and compensatory growth rate after the 4 weeks of dietary manipulation, plus tank as a random effect. Table S3. Mixed model analyses of time required by male sticklebacks to build a nest in relation to age (first breeding or second breeding), season of experiment (Winter or Spring), dietary (restricted or control) and photoperiod (ambient or delayed) treatments, manipulated fish length (at the end of the dietary manipulation, ln transformed) and compensatory growth rate after the 4 weeks of dietary manipulation, plus tank as a random effect. Table S4. No. of eggs in 1st clutch and mean mass of an egg from that clutch in relation to season of experiment (Winter or Spring), dietary (restricted or control) and photoperiod (ambient or delayed) treatment, length at the time of spawning (ln transformed) and compensatory growth rate after the 4 weeks of dietary manipulation in the Winter and Spring experiments. Table S5. Proportion that the eggs produced in the first breeding season made up of the total number of eggs produced by a female over both the first and second breeding seasons, in relation to season of experiment (Winter or Spring), dietary (restricted or control), photoperiod (ambient or delayed), length at time of spawning (ln transformed) and compensatory growth after the 4 weeks of dietary manipulation in the Winter and Spring experiments. Table S6. Maximum lifespan (defined as th [file FEC-30-625-s002.pdf]

Functional Ecology

Functional Ecology 2015 doi: 10.1111/1365-2435.12538

Perturbations in growth trajectory due to early diet affect age-related deterioration in performance

Who-Seung Lee*,†, Pat Monaghan and Neil B. Metcalfe

Institute of Biodiversity, Animal Health and Comparative Medicine, College of Medical, Veterinary and Life Sciences, University of Glasgow, Graham Kerr Building, Glasgow G12 8QQ, UK

Supporting information

Text S1. Additional methods

DETAILS OF THE TIME PERIODS

Period 3 for the first breeding season started on 16 May 2008 for the Winter and 3 July 2008 for the Spring experiment, and Period 5 for the second breeding season started on 6 May 2009 for the ambient photoperiod treatment and 1 June 2009 for the delayed photoperiod treatment). Period 4 was the non-breeding phase between the first and second breeding seasons, when the temperature was again reduced to 10°C. The start of the breeding season was defined as the time when males started to develop their breeding coloration (= reddish throats) and females became gravid (see Table S1 and Lee, Monaghan & Metcalfe 2012 for further details).

QUANTIFICATION OF SWIMMING ENDURANCE

We conducted the swimming trials inside a temperature-controlled room that maintained the temperature the same as in the holding tanks. One fish at a time was placed into a cylindrical swimming chamber (50 cm long, 20 cm diameter). To adapt to the apparatus, the fish was initially subjected for 5 min to a lower velocity of water (17.0 cm s^{-1}). We then changed the water velocity to 34.9 cm s^{-1} (judged to be slightly greater than the maximum that would be sustained by any sticklebacks based on pilot trials) and recorded time until fatigue. Fish were deemed to be exhausted when they were forced back against the fine mesh grid at the

downstream end of the compartment for more than 5 s (Ryan 1988) and were no longer able to continue swimming, despite our tapping the side of the chamber (Ojanguren & Braña 2000). Once fish were exhausted, we immediately turned off the pump and allowed the fish 5 min of recuperation time before being placed back in its original tank. All fish recovered quickly and were swimming normally again within 2-5 min of the pump being switched off.

BREEDING EXPERIMENTAL PROTOCOL

Once males had been placed in their own tank, we added a Petri dish containing fine sand (i.e., a nesting dish) and nesting material (50×5 cm-long threads) in order to allow them to build a nest. A gravid female enclosed in a Plexiglas container was shown to a male for 5 min twice daily for 4 weeks to prompt full expression of nuptial coloration (Pike et al. 2007; Lee, Monaghan & Metcalfe 2012). The same procedures were repeated in the second breeding season.

Text S2. Additional results

COMPENSATORY GROWTH ANALYSIS

As with the analysis of length, mean wet mass of R fish at the end of the manipulation period of the Winter experiment (= manipulated mass) was significantly lighter than that of C fish ($F_{1,9.17}$=109.01, P<0.001), while there was no effect of photoperiod treatment on the mass ($F_{1,8.84}$=0.64, P=0.445). While manipulated mass did not differ between sexes ($F_{1,56.37}$=0.76, P=0.386), there was a significant interaction between dietary regime and sex ($F_{1,57.85}$=7.25, P=0.009): males were lighter in the restricted diet groups while heavier in the control groups. As with fish length, manipulated mass was positively related to body mass at the beginning of the Winter experiment ($F_{1,51.18}$=521.64, P<0.001).

While the manipulated mass was unaffected by photoperiod treatment ($F_{1,10.86}$=1.86, P=0.200) and did not differ between males and females ($F_{1,66.87}$=0.66, P=0.420), as expected there was a significant effect of the dietary treatment ($F_{1,12.66}$=263.72, P<0.001). Manipulated mass in this experiment was also positively related to initial mass ($F_{1,62.37}$=1919.56, P<0.001).

RED THROAT COLORATION AND NEST BUILDING IN MALES

The duration for which males maintained red throat coloration was longer in the first breeding season than in the second, and longer in the Winter experiment than in the Spring (Table S2 and Fig. S3). Dietary treatment affected the duration of red throat coloration (i.e. R males were red for a shorter period of time). While there was no overall effect of photoperiod, there was an interaction between age and photoperiod (the decline in redness with age being much less pronounced in the delayed photoperiod, Table S2, Fig. S3). There was no effect of compensatory growth rate ($F_{1, 51.23}$=0.055, P=0.816), but a male's length at the end of the period of dietary manipulation (= manipulated fish length) was positively related to the length of time he remained red and there was also an interaction between age and manipulated fish length (Table S2), the effect of fish size being more pronounced in the first breeding season.

There was a significant difference in the rate of nest building between the first and the second breeding season, and between the Winter and Spring experiment (Table S3). Males completed nests within 3.3±0.2 days of receiving nest material in their first breeding season (Winter: 3.4±0.3 days and Spring: 3.1±0.2 days) but took longer (4.0±0.3 days) in their second breeding season (Winter: 4.0±0.3 days and Spring: 3.9±0.6 days), and males from the Winter experiment took longer than those from the Spring experiment. While there were no overall effects of diet ($F_{1, 53.08}$=0.71, P=0.405) or photoperiod ($F_{1, 57.71}$=0.02, P=0.891) on the rate of nest building, there was an interaction between season and diet (Table S3): R males took longer than C males to complete nests in the Spring experiment whereas there was less of an effect of diet treatment (after controlling for growth rate – see below) in the Winter experiment (Fig. S4). There was a negative effect of manipulated fish length on duration, plus a significant interaction between age and manipulated fish length (Table S3): the larger the male at the end of the period of dietary manipulation, the shorter the time he took to build a nest. Compensatory growth rate negatively affected the rate of nest building and there was a significant interaction between season and compensatory growth rate (Table S3): the faster the compensatory growth rate, the longer the time needed to build a nest, particularly in the Spring experiment.

REPRODUCTIVE INVESTMENT IN FEMALES

A total of 24 and 25 females (out of 29 and 35 that were alive at the time) spawned during the first breeding season in the Winter and Spring experiments respectively, but only 9 females in

the Winter experiment and 7 in the Spring experiment spawned in the second season (out of 23 and 31 that were still alive at that time). Given the low numbers of females spawning in the second season, the analysis of reproductive investment is based primarily on the first breeding season, and analysis of individual egg mass and number of eggs per clutch is only based on the first clutch since the number of clutches varied between females (mean (±standard deviation) number of clutches per female in the first season = 1.26±0.65). The mean mass per egg from the 1st clutch of each female was significantly heavier in the Winter experiment (3.3±0.1 mg) than in the Spring (2.4±0.2 mg; Table S4). While there was no effect of compensatory growth ($F_{1,31.72}$=2.54, P=0.121), the mass of an egg was related to a female's length at the time of spawning (Table S4), with larger fish producing heavier eggs. Dietary treatment also affected egg mass (with R females of a given size producing lighter eggs, Table S4) whereas there was no effect of photoperiod treatment ($F_{1,21.76}$=0.53, P=0.475). Egg mass was affected by interactions between season and diet and between diet and length at time of spawning (Table S4): the effect of diet was strongest in the Spring experiment, and females from the R group showed less of an effect of fish size on egg size (Fig. S5a and c).

The number of eggs in the first clutch was not significantly different between the Winter (63.6±2.8) and Spring experiments (52.2±5.5) whereas there was an effect of dietary treatment (Table S4), with R fish producing fewer eggs than C fish (Fig. S5b and d). Females from the delayed photoperiod group spawned more eggs than those under an ambient photoperiod (Table S4, Fig. S5b and d). As with egg size, there was no effect on clutch size of compensatory growth ($F_{1, 32.25}$=0.55, P=0.465) but a positive effect of the female's length at time of spawning (Table S4 and Fig. S5b and d). The interaction between season and photoperiod significantly affected the number of eggs, with delayed photoperiod fish in the Winter experiment spawning more eggs (Table S4).

The relative investment in the first breeding season (defined as (the total number of eggs a female produced in Period 3) / (combined number of eggs she produced in Periods 3 and 5)) was analysed to understand how growth trajectories influenced the investment by the female over time. There were significant differences between the Winter and Spring experiments in the proportion that the first season's eggs made up of the total egg production in the two years (Table S5), with females from the Spring experiment showing a greater relative investment in the first season (Fig. S6). While there was no effect of diet (Table S5) or photoperiod ($F_{1, 39}$=0.01,

P=0.919), there was a significant interaction between season and diet (Table S5): R females in the Spring experiment invested relatively less in egg production in the second breeding season than did the corresponding females in the Winter experiment (Fig. S6). While there was no effect of length at time of first spawning ($F_{1, 39}$=3.68, P=0.062), compensatory growth rate positively affected the proportion of eggs produced in the first year (Table S5).

MAXIMUM LIFESPAN

The maximum lifespan (defined as the age at which 90% of the population had died) was similar to the median lifespan (Table S6): in both Winter and Spring experiments, and in both photoperiod treatments, the maximum lifespan of R fish was shorter than that of C fish (with an average reduction in maximum lifespan over all treatment groups of 9.9±3.3 (s.e.) %). Fish under a greater time stress had a reduced maximum lifespan (with an average reduction of 17.8±2.2% when comparing equivalent treatment groups in the Spring vs. Winter experiments, and an average reduction of 12.4±3.6% when comparing equivalent treatment groups in the Ambient vs. Delayed photoperiods; Table S6).

Supplemental references

Lee, W.-S., Monaghan, P. & Metcalfe, N.B. (2012) The pattern of early growth trajectories affects adult breeding performance. *Ecology*, **93,** 902-912.

Lee, W.-S., Monaghan, P. & Metcalfe, N.B. (2013) Experimental demonstration of the growth rate - lifespan trade-off. *Proceedings of the Royal Society B-Biological Sciences*, **280,** 20122370.

Ojanguren, A.F. & Braña, F. (2000) Thermal dependence of swimming endurance in juvenile brown trout. *Journal of Fish Biology*, **56,** 1342-1347.

Pike, T.W., Blount, J.D., Bjerkeng, B., Lindstrom, J. & Metcalfe, N.B. (2007) Carotenoids, oxidative stress and female mating preference for longer lived males. *Proceedings of the Royal Society B-Biological Sciences*, **274,** 1591-1596.

Ryan, M.J. (1988) Phenotype, genotype, swimming endurance and sexual selection in swordtail (*Xiphophorus-Nigrensis*). *Copeia*, **1988,** 484-487.

Table S1. Description of experimental manipulations. Note that during Period 1 Restricted (R) fish were fed a restricted diet (2% of body mass) while Control (C) fish were fed *ad libitum*. After Period 1, all fish were fed *ad libitum*. Temperature was held at 10°C during Periods 1, 2 and 4, but was increased to 14°C during the breeding periods in 2008 and 2009 (Period 3 and 5). These manipulations were conducted on separate fish in the Winter and Spring experiment.

	Dietary manipulation		Photoperiod
Group	Period 1	Period 2 to 5	manipulation
R Ambient	Restricted (2% of body mass)	*Ad libitum* food ration	Ambient
C Ambient	*Ad libitum* food ration	*Ad libitum* food ration	Ambient
R Delayed	Restricted (2% of body mass)	*Ad libitum* food ration	Delayed (35 days)
C Delayed	*Ad libitum* food ration	*Ad libitum* food ration	Delayed (35 days)

Table S2. Mixed model analyses of red throat colouration of male sticklebacks in relation to age (first breeding or second breeding), season of experiment (Winter or Spring), dietary (restricted or control) and photoperiod (ambient or delayed) treatments, manipulated fish length (at the end of the dietary manipulation, ln transformed) and compensatory growth rate after the 4 weeks of dietary manipulation, plus tank as a random effect. Non-significant variables were dropped from the final model.

Final model	*F*	df	*P*
Age	10.18	1, 38.57	0.003
Season	4.81	1, 44.43	0.034
Dietary	49.39	1, 41.37	<0.001
Photoperiod	3.28	1, 45.09	0.077
Manipulated fish length	6.70	1, 45.99	0.013
Age × photoperiod	8.96	1, 35.62	0.005
Age × manipulated fish length	9.61	1, 39.06	0.004

Table S3. Mixed model analyses of time required by male sticklebacks to build a nest in relation to age (first breeding or second breeding), season of experiment (Winter or Spring), dietary (restricted or control) and photoperiod (ambient or delayed) treatments, manipulated fish length (at the end of the dietary manipulation, ln transformed) and compensatory growth rate after the 4 weeks of dietary manipulation, plus tank as a random effect. Non-significant variables were dropped from the final model.

Final model	*F*	df	*P*
Age	10.72	1, 35.66	0.002
Season	12.63	1, 54.29	0.001
Dietary	0.71	1, 53.08	0.405
Manipulated fish length	13.44	1, 65.04	<0.001
Compensatory growth rate	5.16	1, 58.78	0.027
Season × dietary	6.63	1, 54.05	0.013
Season × compensatory growth rate	10.53	1, 59.72	0.002

Table S4. No. of eggs in 1st clutch and mean mass of an egg from that clutch in relation to season of experiment (Winter or Spring), dietary (restricted or control) and photoperiod (ambient or delayed) treatment, length at the time of spawning (ln transformed) and compensatory growth rate after the 4 weeks of dietary manipulation in the Winter and Spring experiments. The LME included season, diet and photoperiod as fixed effects, fish length at spawning and compensatory growth rate after 4 weeks manipulation as covariates and tank as random effects, plus all interactions. Non-significant variables were dropped from the final model.

	Final model	*F*	df	*P*
Mass of each egg	Season	37.55	1, 30.00	<0.001
	Dietary	4.98	1, 38.37	0.032
	Length at time of spawning	28.74	1, 38.40	<0.001
	Season × dietary	7.76	1, 21.00	0.011
	Dietary × length at time of spawning	6.13	1, 38.40	0.018
No. of eggs in 1st clutch	Season	1.77	1, 21.92	0.197
	Dietary	5.89	1, 21.59	0.024
	Photoperiod	6.89	1, 21.58	0.016
	Length at time of spawning	10.72	1, 38.05	0.002
	Season × photoperiod	4.64	1, 21.09	0.043

Table S5. Proportion that the eggs produced in the first breeding season made up of the total number of eggs produced by a female over both the first and second breeding seasons, in relation to season of experiment (Winter or Spring), dietary (restricted or control), photoperiod (ambient or delayed), length at time of spawning (ln transformed) and compensatory growth after the 4 weeks of dietary manipulation in the Winter and Spring experiments. The LME included season, diet and photoperiod as fixed effects, fish length at spawning and compensatory growth rate after 4 weeks manipulation as covariates and tank as random effects, plus all interactions. Non-significant variables were dropped from the final model.

Final model	*F*	df	*P*
Season	9.35	1, 40	0.004
Dietary	2.73	1, 40	0.106
Compensatory growth rate	6.50	1, 40	0.015
Season × dietary	4.20	1, 40	0.047

Table S6. Maximum lifespan (defined as the age by which 90% of the population in a treatment group had died) in relation to dietary and photoperiod treatments. Also shown is the % difference compared to the value for control fish of the same photoperiod and season of experiment; note that negative values in % difference compared to control value presents decrease in maximum lifespan relative to the corresponding control fish.

	Treatment			
Season of experiment	Photoperiod	Dietary	Maximum lifespan (days)	% difference compared to control value
Winter	Ambient	Restricted	966	-6.76
		Control	1036	
	Delayed	Restricted	1032	-12.54
		Control	1180	
Spring	Ambient	Restricted	781	-2.62
		Control	802	
	Delayed	Restricted	852	-17.84
		Control	1037	

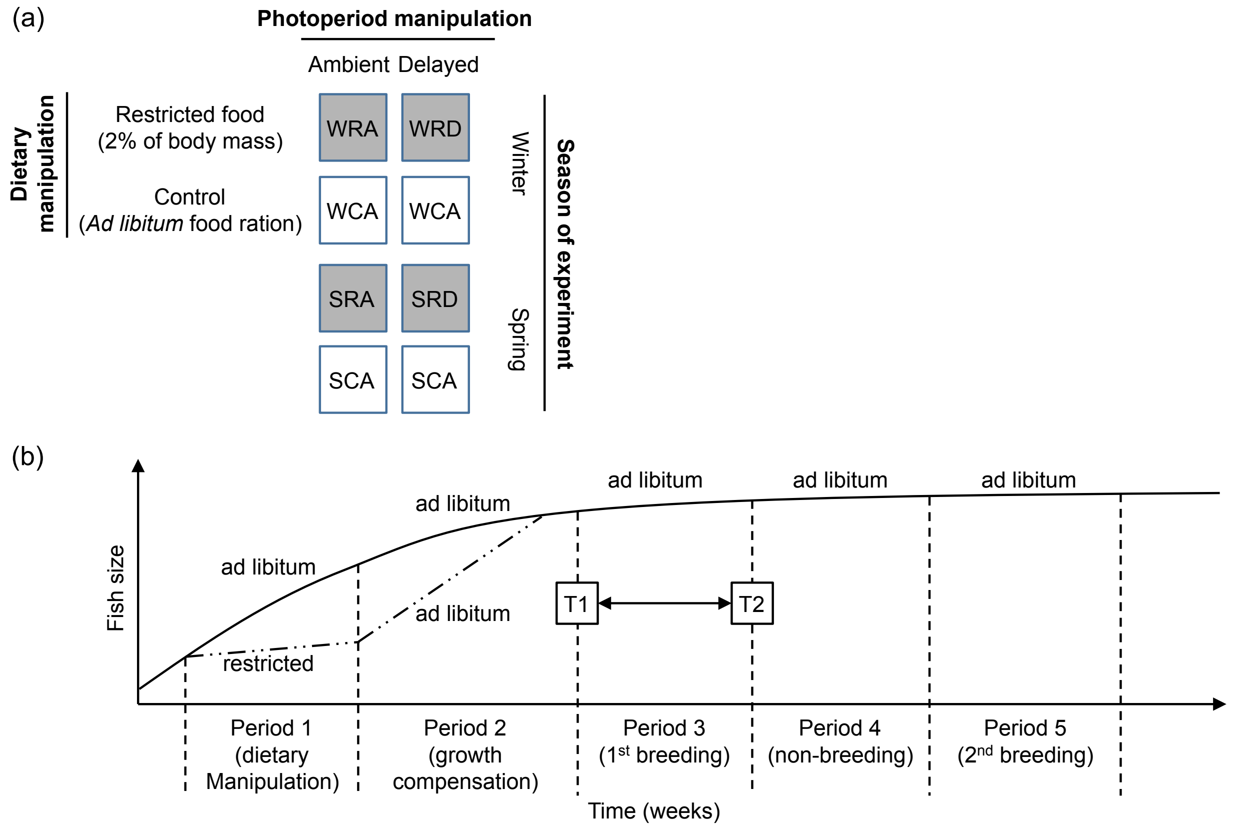

Fig. S1. (*a*) Illustration of the experimental design, with three treatments (dietary, photoperiod and season of experiment). R, restricted food manipulation, fed 2% of body mass during Period 1; C, control group, fed *ad libitum*; A, ambient photoperiod treatment; D, delayed photoperiod treatment; W, winter experiment; S, spring experiment. (*b*) Description of experimental schedule, with illustration of growth trajectories of two dietary treatment groups (restricted food treatment – double dashed line, control – solid line): Period 1 – the 4 week dietary manipulation period during which R fish were fed the restricted diet while Control (C) fish were fed *ad libitum*. After Period 1, all fish were fed *ad libitum*. Period 2 – the compensatory period, Period 3 – the first breeding season, Period 4 – non-breeding season, Period 5 – the second breeding season. 'T1' and 'T2' indicate the timing of swimming trials (i.e., at the end of the period of compensatory growth and 18 weeks later, after the breeding season).

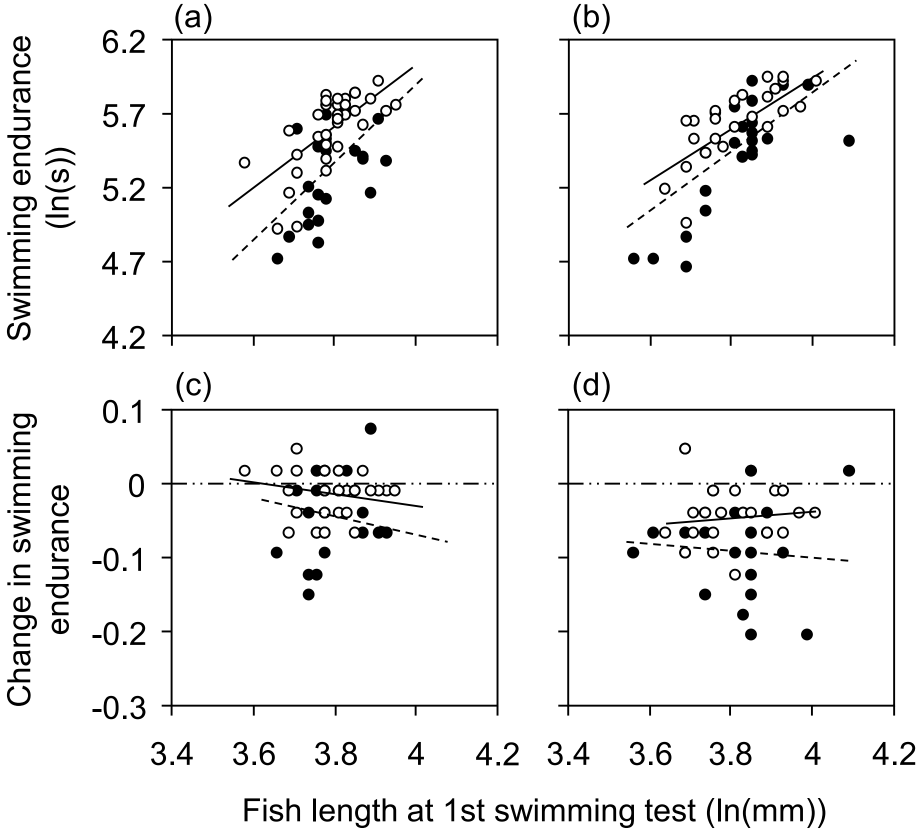

Fig. S2. Effects of dietary treatment on swimming performance in three-spined sticklebacks: (*a* and *b*) swimming endurance (ln(s)) at the end of the compensatory period in relation to fish length at the time (ln(mm)) and (*c* and *d*) change in swimming endurance over the breeding season (as the amount of the advance in the second trial compared to the first trial, see Materials and Methods for a formula) in relation to fish length at time of first swimming test. Individual data points and regression lines are plotted from the Winter (left panel) and Spring (right panel) experiments, categorised by dietary treatment (restricted: black circle and dashed line, control: open circle and solid line). Data are plotted according to dietary treatment and experiment as in Fig. S1.

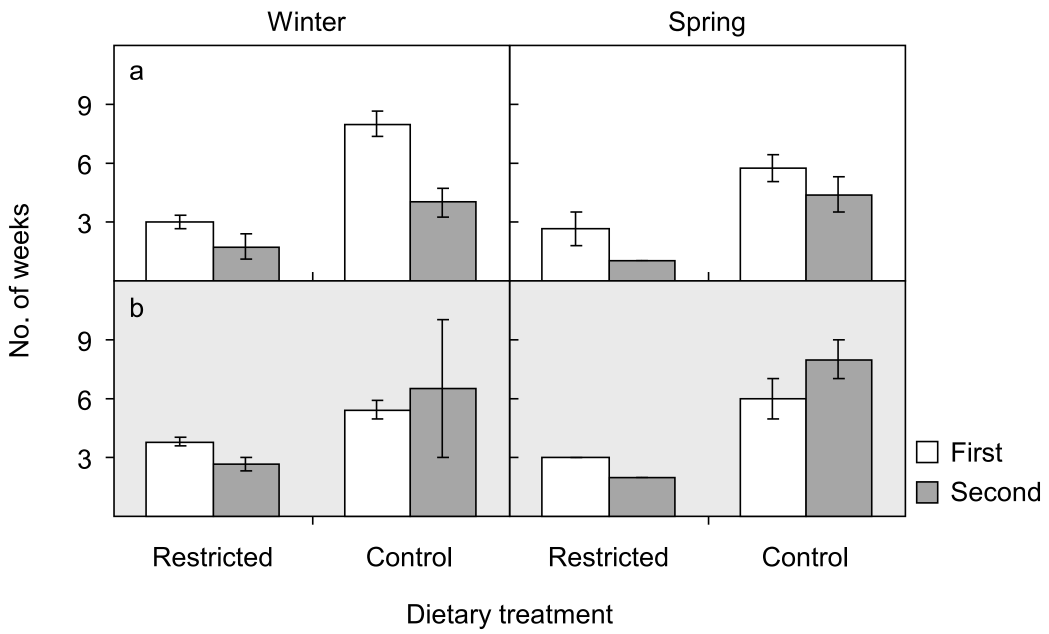

Fig. S3. No. of weeks that male three-spined sticklebacks maintained a strong red throat colour (i.e. exceeding the population mean score) in their first (white bar) and second (grey bar) breeding season, in relation to dietary manipulation (restricted or control) and photoperiod regime ((*a*) ambient or (*b*) delayed) in both the Winter (left panel) and Spring (right panel) experiments. Data plotted as means ± SE. See Table S2 for statistical analysis.

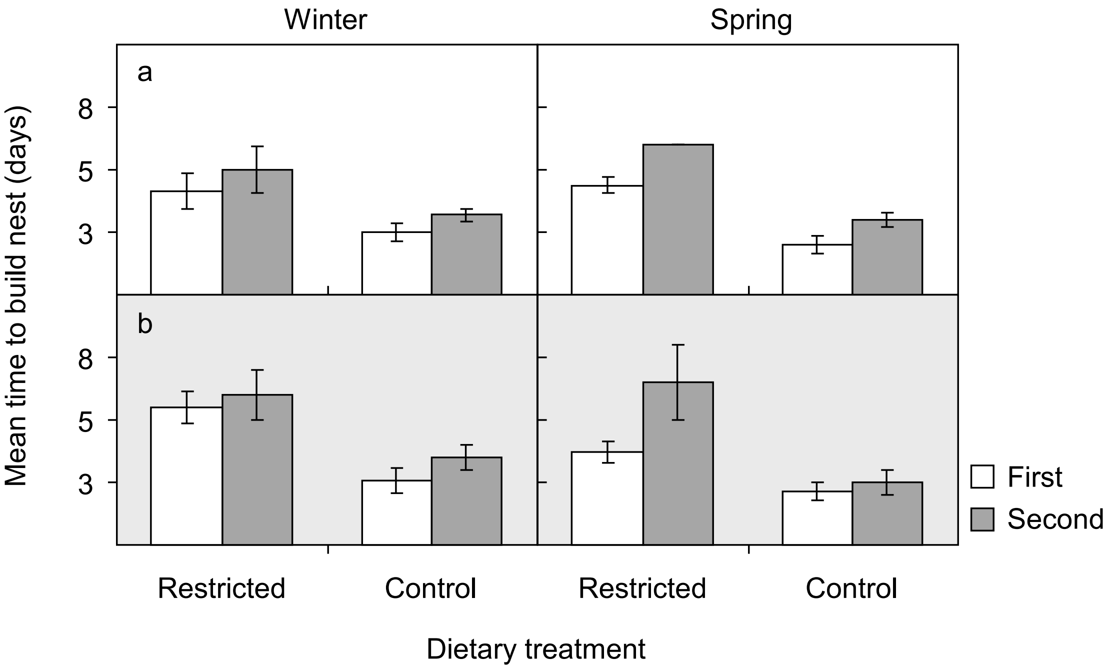

Fig. S4. Time taken by male three-spined sticklebacks to build a nest (days, mean ± SE) in relation to dietary manipulation (restricted or control) and photoperiod manipulation ((*a*) ambient or (*b*) delayed) in both the Winter (left panel) and Spring (right panel) experiments. Data plotted as means ± SE. See Table S3 for statistical analysis.

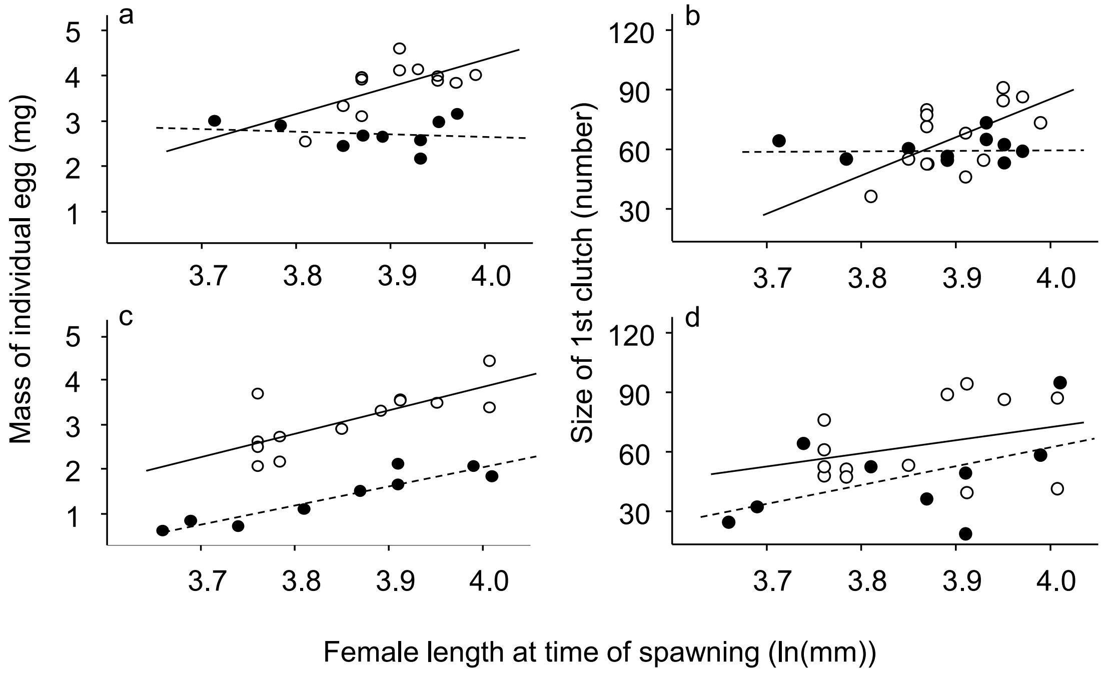

Fig. S5. Mean mass of individual eggs (mg, *a* and *c*) from the first clutch and size of the first clutch (number of eggs, *b* and *d*) produced by one year old female three-spined sticklebacks during the first breeding period in relation to their length at the time of spawning (mm, ln transformed). Values are plotted separately for the two dietary manipulation treatment groups (restricted – black circle and dashed line; control – open circle and solid line) in the Winter (*a* and *b*) and Spring (*c* and *d*) experiments.

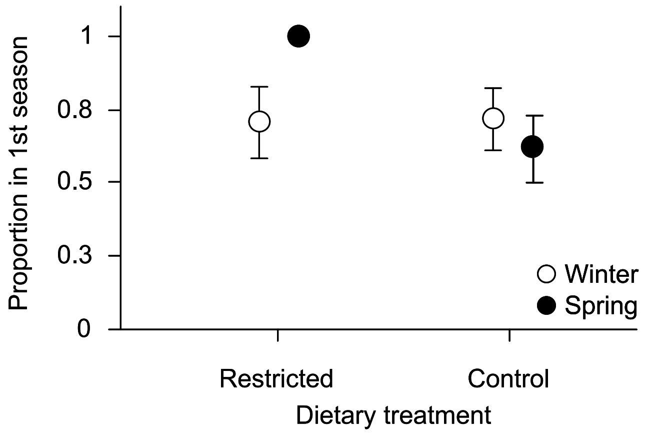

Fig. S6. Proportion that the eggs produced in the first breeding season made up of the total number of eggs produced by a female over both the first and second breeding seasons. Data shown in relation to dietary treatment (restricted or control) and experiment (Winter or Spring); data plotted as means±SE.
